# Overexpression of *NtPR-Q* Up-Regulates Multiple Defense-Related Genes in *Nicotiana tabacum* and Enhances Plant Resistance to *Ralstonia solanacearum*

**DOI:** 10.3389/fpls.2017.01963

**Published:** 2017-11-16

**Authors:** Yuanman Tang, Qiuping Liu, Ying Liu, Linli Zhang, Wei Ding

**Affiliations:** College of Plant Protection, Southwest University, Chongqing, China

**Keywords:** *NtPR-Q*, *Nicotiana tabacum*, *Ralstonia solanacearum*, plant resistance, pathogenesis-related proteins

## Abstract

Various classes of plant pathogenesis-related proteins have been identified in the past several decades. PR-Q, a member of the PR3 family encoding chitinases, has played an important role in regulating plant resistance and preventing pathogen infection. In this paper, we functionally characterized *NtPR-Q* in tobacco plants and found that the overexpression of *NtPR-Q* in tobacco Yunyan87 resulted in higher resistance to *Ralstonia solanacearum* inoculation. Surprisingly, overexpression of *NtPR-Q* led to the activation of many defense-related genes, such as salicylic acid (SA)-responsive genes *NtPR1a/c*, *NtPR2* and *NtCHN50*, JA-responsive gene *NtPR1b* and ET production-associated genes *NtACC Oxidase* and *NtEFE26*. Consistent with the role of *NtPR-Q* in multiple stress responses, *NtPR-Q* transcripts were induced by the exogenous hormones SA, ethylene and methyl jasmonate, which could enhance the resistance of tobacco to *R. solanacearum*. Collectively, our results suggested that *NtPR-Q* overexpression led to the up-regulation of defense-related genes and enhanced plant resistance to *R. solanacearum* infection.

## Introduction

Plants have a two-branched immune system when responding to the microbial infection. The first branch uses pattern recognition receptors (PRR) to recognize molecules common to many classes of microbes, generally known as PAMP-triggered immunity (PTI). The second layer of the immune response uses NB-LRR (nucleotide binding and leucine rich repeat) proteins which are encoded by most *R* genes to recognize pathogen effector, also known as effector-triggered immunity (ETI) (Jones and Dangl, 2006). Plant resistance refers to the ability of plants to overcome or inhibit the effect of a pathogen or other damaging environment factors to a certain extent (Agrios, 1988). Plants respond to external environmental stress through rapid changes in resistance gene expression, resulting in producing a number of specific proteins, including resistance-related proteins (RRP) and resistance-related metabolites (RRM), which are either only present in resistant genotypes or are found in higher accumulation in the resistant genotype than in the susceptible genotype (Kushalappa et al., 2016). It is a common strategy to improve plant resistance by constructing transgenic plants that overexpress transcription factors (Dang et al., 2013; Wang et al., 2013; Jia et al., 2015) or pathogenesis-related genes. Phytohormones, such as salicylic acid (SA), jasmonic acid (JA), and ethylene (ET), act as signal molecules during plant growth and stress response, and their content and activity in plant are highly susceptible to changes of external conditions and cell growth. It is found that SA-dependent responses act against biotrophs pathogens, and JA/ET-dependent responses act against necrotrophs pathogens (Glazebrook, 2005; Vlot et al., 2009).

The pathogenesis-related (PR) proteins are a class of proteins that suppress pathogens, detoxify toxins or virulence factors produced by pathogens and prevent pathogen advancement by enforcing cell walls (Kushalappa et al., 2016). Pathogenesis-related proteins have attracted an increasing research interest in view of their possible involvement in plant resistance to pathogens. There is a close relationship between PRs and SAR (Payne et al., 1990), and they have been considered as a marker of the induced state (Ward et al., 1991b; Uknes et al., 1992; Kessmann et al., 1994). Thaumatin-like protein (TLP-3) has been reported to enhance resistance to *Alternaria alternata* in transgenic tobacco plants (Safavi and Zareie, 2012). A pepper gene, CABPR1, which encodes PR1, has been reported to be strongly induced by wounding, ethephon treatment, and tobacco mosaic virus (TMV) infection (Sarowar et al., 2005). PRs have been classified into 17 families, and the PR-3 family has chitinase properties (Sels et al., 2008). Chitinases can be divided into two categories: exochitinases and endochitinases, and PR-Q belongs to the latter category (Payne et al., 1990). Many plant endochitinases exhibit an additional lysozyme or lysozyme-like activity. It is known that chitinases have significant antifungal activities against pathogenic fungi, such as *Fusarium oxysporum* and *Cercospora nicotianae* (Zhu et al., 1994; Jongedijk, 1995). PR-3 proteins can cleave the cell wall chitin polymers *in situ*, so that the cell wall weaken and enhance osmotic sensitivity to fungi. The expression of chitinases is regulated by wounding, methyl jasmonate (MeJA), ethylene, and gibberellin (Golshani et al., 2015). In tomato, the simultaneous expression of tobacco chitinase gene *PR-3d* and glucanase gene *PR-2e* lead to stronger resistance to *F. oxysporum* f.sp. *lycopersici*, whereas transgenic plants expressing either one of these genes are susceptible (Jongedijk, 1995). PR-Q, a member of the PR-3 family, is a class II chitinase. The acidic enzyme is found in the extracellular fluid from leaves, suggesting that it is localized in the apoplastic compartment (Payne et al., 1990; Ohme-Takagi et al., 1998). It is part of the systemic resistance and preventing pathogen infection.

As a complex species (Prior and Fegan, 2005), *Ralstonia solanacearum* has complex genetic diversity and strong adaptability to the environment (Horita et al., 2010; Liu et al., 2017b). *R. solanacearum* causes wilting symptoms to a variety of crops, including vegetables (*Solanum tuberosum*, *S. lycopersicum*, *Capsicum* spp.), tobacco (*Nicotiana tabacum*), peanuts (*Arachis hypogaea*), banana (*Musa* spp.), and so on (Ji et al., 2005). Since the first report of bacterial wilt appeared in 1864, the disease, resulting in serious economic losses, has been found in major countries and regions worldwide where *Solanaceae* crops are cultivated. At present, the control of bacterial wilt mainly depends on chemical pesticides (Yang et al., 2016), but based on the strong variability and soil-borne characteristics of *R. solanacearum*, the challenge for actual crop production remains considerable. It is important to study the functions of disease-resistance genes to improve plant resistance.

In this study, the *NtPR-Q* gene was overexpressed in tobacco, and its biological function in defending against *R. solanacearum* was analyzed. This study provides new information for the disease control network and a new strategy for disease resistance breeding.

## Materials and Methods

### Plant Materials and Growth Conditions

A *N. tabacum* cultivar, Yunyan87, was used in this study as the wild type (WT-Yunyan87). The surface of the seeds were disinfected with 75% alcohol for 30 s and 10% H_2_O_2_ for 10 min, then washed five times with sterile water and placed under greenhouse conditions for 2–3 weeks on MS agar medium (Murashige and Skoog, 1962; Dang et al., 2013). Regenerated shoots and healthy resistant shoots were grown on selective shooting medium and rooting medium, respectively, which both contained 500 mg⋅L^-1^ carbenicillin and 100 mg⋅L^-1^ kanamycin. The transgenic plants for this study were obtained by asexual reproduction. Well-developed rooted plants were transferred to matrix in plastic pots and then placed under greenhouse conditions at 25 ± 2°C with a relative humidity of 80% and 16/8 h light/dark cycle.

### Pathogens and Inoculation Procedures

*Ralstonia solanacearum* CQPS-1 (phylotype I, race 1, biovar 3) (Liu et al., 2017a) was maintained in our laboratory and was isolated from Chongqing Municipality, China. *R. solanacearum* strains were cultured at 180 rpm, 30°C in NB medium (3 g⋅L^-1^ beef extract, 1 g⋅L^-1^ yeast extract, 5 g⋅L^-1^ peptone and 10 g⋅L^-1^ glucose), and bacterial suspension was cultured to about 1 × 10^9^ CFU mL^-1^.

To study the relative expression levels of *NtPR-Q* in response to *R. solanacearum* infections, WT-Yunyan87 plants were inoculated with 10 mL of bacterial suspension (1 × 10^8^ CFU mL^-1^). Leaf samples were harvested at 1, 3, 5, and 7 days post inoculation (dpi), frozen and stored at -80°C until further analysis. Control plants were inoculated with sterile water. Three individual plants were mixed to yield one sample.

For the pathogenicity assays, transgenic plants and WT-Yunyan87 plants were root inoculated with a 10 mL bacterial suspension (1 × 10^8^ CFU mL^-1^). Assays were conducted with 10 plants and were repeated three times. The disease index of bacterial wilt were surveyed every day for 30 days using a 0 to 4 grading standards, where 0 = asymptomatic plant, 1 = less than 25% leaves wilted, 2 = less than 50% wilted leaves, 3 = more than 50% wilted leaves and 4 = completely wilted leaves (dead plant). The data presented with the average disease index (Wang et al., 2013).

For monitoring a bacterial growth assay in leaf tissues, bacterial suspension (5 × 10^7^ CFU mL^-1^) was washed twice with sterile water and resuspended in sterile water. Transgenic plants and WT-Yunyan87 plants were inoculated by infiltrating 20 μL inoculum into the third leaves from the top using a blunt syringe. The inoculated leaves were harvested at the indicated time points (30 min, 1, 3, and 5 days) using a leaf punch 1 cm in diameter and were then grounded, serially diluted, and spread on TTC medium [3 g⋅L^-1^ beef extract, 1 g⋅L^-1^ yeast extract, 5 g⋅L^-1^ peptone, 10 g⋅L^-1^ glucose, 20 g⋅L^-1^ agar configured to NA medium, 100 mL NA medium supplemented with 500 μL triphenyl tetrazolium chloride (TTC) before use]. The number of colonies was counted after 2 days, and the number of *R. solanacearum* cells per square centimeter of leaves was calculated. The assay repeated three times.

To test the expression of defense-related genes in transgenic tobacco, we harvested the leaf of transgenic tobacco and WT-Yunyan87 tobacco which were grown on MS medium. To study the relative expression levels of defense-related genes in response to *R. solanacearum* infection, transgenic plants and WT-Yunyan87 plants were inoculated with 10 mL bacterial suspension (1 × 10^8^ CFU mL^-1^). Leaf samples were harvested at 1, 3, and 5 dpi, frozen and stored at -80°C until further analysis. Control plants were inoculated with sterile water. Three individual plants were mixed to yield one sample.

### Plant Treatment with Exogenous Hormones

WT-Yunyan87 tobacco plants at the four-leaf stage were sprayed with 0.1 mM MeJA, 2 mM SA, or 7 mM ethephon. SA was dissolved in 10% ethanol, and the others were dissolved in sterile water. Control plants were sprayed with 10% ethanol and sterile water. The disease index of bacterial wilt were surveyed every day for 26 days using 0 to 4 grading standards. Assays were conducted with 10 plants and were repeated three times. Plants treated with SA, MeJA, or ethephon were also harvested as samples at 3, 6, 12, and 24 h post treatment, frozen and stored at -80°C for the quantitative real-time PCR analysis. Three individual plants were mixed to yield one sample.

### RNA Extraction, cDNA Synthesis

The leaves of plants were grounded into powder in liquid nitrogen using mortars. RNA was extracted using an RNAprep Pure Plant Kit (Tiangen, DP432, Beijing, China). The RNA sample was then reverse transcribed with an iScript^TM^ cDNA Synthesis Kit (BIO-RAD, 1708891, Hercules, CA, United States) in a 20 μL reaction.

### Quantitative Real-Time PCR

To analyze the relative transcript levels of various genes, quantitative real-time PCR (RT-qPCR) was performed with specific primers, *NtUBI3* as the reference gene (Zhao et al., 2009) (see **Table 1** for the gene-specific primers) using SsoFast^TM^ EvaGreen^®^ Supermix (BIO-RAD, 1725201, Hercules, CA, United States) and the CFX96 real-time system (BIO-RAD, United States). The 20 μL reaction mixtures contained 1 μL cDNA, 10 μL SsoFast^TM^ EvaGreen^®^ Supermix, 1 μL of each gene-specific primer (10 μM) and 7 μL ddH_2_O. The amplifications were performed at 95°C for 3 min, followed by 40 cycles of denaturation at 95°C for 15 s, 55°C for 20 s and elongation at 56°C for 30 s. Melt curve analyses at 95°C were included at the end to ensure the consistency of the amplified products. The expression level and data analyses were performed by using Bio-Rad CFX Manager 3.0 software with the ^ΔΔ^*C*q method (Li et al., 2017). Each assay was performed with three independent technical repeats and the expression level of the genes was calculated from three biological replicates.

**Table 1:** Main primers for PCR.

Gene	Accession number	Forward primer (5′–3′)	Reverse primer (5′–3′)
*NtPR-Q* vector	CAA35789.1	GGAAGATCTATGGAGTTTTCTGGATCACC	GGGTAACCTTATTATTAATGATGATGATGA TGATGGCCCTGGCCGAAGTTCCT
*NtPR-Q* RT-qPCR	CAA35789.1	GCACAAGGCATTGGTTCTA	TGGTTCTGCACTCAGGGAT
*NtPR-Q* insertion	CAA35789.1	ATGACGCAATCCCACTATCC	GCCCTGGCCGAAGTTCCT
*NtHIN1*	Y07563	CGACCTAACAAAGTCAAGTTCTACG	CTCTATCTCCCAATAAAACCAAGC
*NtHSR201*	X95343	CAGCAGTCCTTTGGCGTTGTC	GCTCAGTTTAGCCGCAGTTGTG
*NtPR1a/c*	X05959	AACCTTTGACCTGGGACGAC	GCACATCCAACACGAACCGA
*NtPR2*	M60460	TGATGCCCTTTTGGATTCTATG	AGTTCCTGCCCCGCTTT
*NtEFE26*	Z29529	CGGACGCTGGTGGCATAAT	CAACAAGAGCTGGTGCTGGATA
*NtCHN50*	X51599	ATGCCAAGGAAAGGGATTCTACA	TGGGAGGTTTGGGCGAAGA
*NtPR1b*	X66942	AACCCATCCATACTATTCCTTG	GCCGCTAACCTATTGTCCC
*NtACC Oxidase*	AB012857	GACAAAGGGACATTACAAGAAGT	GAGAAGGATTATGCCACCAG
*NtCAT1*	AY128694.1	CAACTTCCTGCTAATGCTCCAA	TGCCTGTCTGGTGTGAATGA
*NtGST1*	D10524	AGCACCCTTACCTTTCCCTC	GCTTTCCTTCACAGCAGCATCA
*NtUBI3*	X58253	GCCGACTACAACATCCAGAAGG	TGCAACACAGCGAGCTTAACC
*NtACTIN*	FM244697	AGGGTTTGCTGGAGATGATG	CGGGTTAAGAGGTGCTTCAG

### Construction of Plant Overexpression Vector and Agrobacterium Transformation

The full-length cDNA of *NtPR-Q* with a downstream in-frame His-Tag was cloned using *Bgl*II and *Bst*EII restriction sites. The specific primers of *NtPR-Q* vector used in this study are listed in **Table 1**. The plant expression vector pVCT2024 was provided by Dr. Xingguo Zhang (College of Horticulture and Landscape Architecture, Southwest University). After digestion with *Bgl*II and *Bst*EII, the *NtPR-Q* cDNA was inserted into pVCT2024 under the control of the *Cauliflower mosaic virus* (CaMV) 35S promoter. The positive recombinant plasmid pVCT2024-*NtPR-Q* was transformed into *Agrobacterium tumefaciens* strain EHA 105, which was then transformed into WT-Yunyan87 plants using the leaf disk method described by Oh et al. (2005). Transgenic plants were further confirmed to have T-DNA insertions by standard PCR and RT-qPCR.

### PCR Analysis of the T-DNA Insertion

To analyze the T-DNA insertion in transgenic tobacco, standard PCR was performed with the primers of *NtPR-Q* insertion and *NtACTIN* (see **Table 1**), WT-Yunyan87 tobacco plants as the blank control, *NtACTIN* served as an endogenous control (Dang et al., 2013). The 20 μL reaction mixtures contained 1 μL cDNA, 2 μL 10 × buffer (TaKaRa, Dalian, China), 1 μL of each gene-specific primer (10 μM), 1.6 μL dNTP (TIANGEN, Beijing, China), 0.1 μL Taq DNA polymerase (TIANGEN, Beijing, China), and 13.3 μL ddH_2_O. The amplifications were performed at 94°C for 5 min, followed by 35 cycles of denaturation at 94°C for 30 s, 59°C for 30 s and elongation at 72°C for 2.5 min, 72°C for 5 min. PCR product was electrophoresed on 1% agarose gel and observed by Bio-Rad gel imager.

### Statistical Analysis

The SPSS version 17.0 software was used for statistical analysis. The data between two treatments were compared and statistically analyzed through the Student *t*-test. The data among multiple treatments were compared and statistically analyzed though one-way analysis of variance and Student–Newman–Keuls test.

## Results

### Response of *NtPR-Q* Transcript Levels to *R. solanacearum* Infection and Hormone Application

**FIGURE 1:**
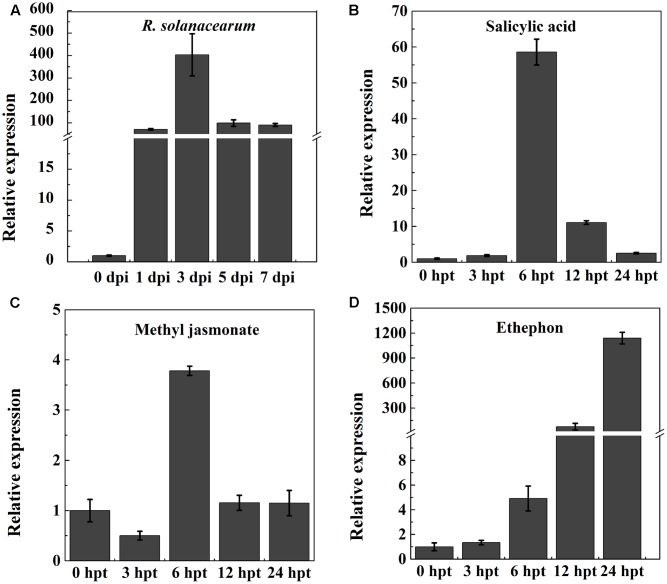
RT-qPCR analysis of the relative *NtPR-Q* transcript levels in WT-Yunyan87 tobacco plants exposed to stress. **(A)** Relative *NtPR-Q* transcript levels measured at different time points in WT-Yunyan87 tobacco after inoculation with *Ralstonia solanacearum*. Plants inoculated with sterile water served as control. **(B–D)** Relative *NtPR-Q* transcript levels measured at different time points in WT-Yunyan87 tobacco after treatment with salicylic acid, methyl jasmonate and ethephon. Control plants were sprayed with 10% ethanol or sterile water. *NtUBI3* as the reference gene. The averages presented are based on three independent biological replicates for each set of plants.

To test whether *NtPR-Q* is involved in the plant immune response against *R. solanacearum*, the transcriptional expression levels of *NtPR-Q* in WT-Yunyan87 was determined by RT-qPCR after inoculation with *R. solanacearum* strain CQPS-1 (**Figure 1A**). Compared with 0 hpi, *NtPR-Q* transcripts levels were enhanced in tobacco leaves starting at 1 dpi, and reached the maximum at 3 dpi. Subsequently, at 5 dpi, the *NtPR-Q* transcript levels decreased to the level of 1 dpi and maintained until 7 dpi. These results showed that transcripts of *NtPR-Q* accumulate in response to inoculation with *R. solanacearum*, suggesting *NtPR-Q* might be involved in defense reactions of tobacco to *R. solanacearum.*

Phytohormones are important signaling molecules that play an important role in the regulation of plant defense reactions against external stresses and mediating the expression of downstream defense genes (Fujita et al., 2006). To evaluate the possible involvement of *NtPR-Q* in the hormone signaling pathways, *NtPR-Q* transcript levels were determined by RT-qPCR in tobacco leaves after exogenous treatments with SA, MeJA, and ethephon. SA application enhanced the *NtPR-Q* transcript levels between 0 and 24 h, reaching the maximum at approximately 6 h (**Figure 1B**). In response to MeJA, the transcript levels of *NtPR-Q* was reduced at 3 h, but increased to the maximal level at 6 h and then returned to the basal level at 24 h (**Figure 1C**). After ethephon treatment, the *NtPR-Q* transcript levels increased between 0 and 24 h (**Figure 1D**). The transcript levels of *NtPR-Q* after the application of ethephon were the highest of the three treatments.

### Overexpression of *NtPR-Q* Enhances the Resistance of Tobacco to *R. solanacearum*

**FIGURE 2:**
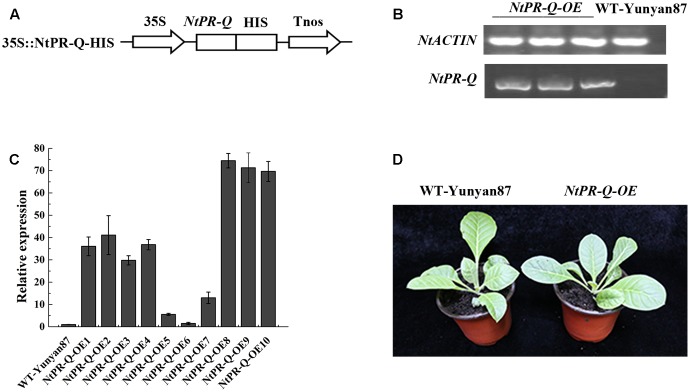
Generation of *NtPR-Q*-overexpressing tobacco plants. **(A)** Schematic diagram of the 35S::NtPR-Q-HIS fusion protein construct. **(B)** PCR analysis of the T-DNA insertion in representative transgenic tobacco plants. WT-Yunyan87 tobacco plants as the blank control, and *NtACTIN* served as an endogenous control. **(C)** Relative transcripts of *NtPR-Q* in *NtPR-Q-OE* lines and WT-Yunyan87 plants were tested by RT-qPCR. The relative transcript levels in WT-Yunyan87 plants as the control, *NtUBI3* as the reference gene. The averages presented are based on three independent biological replicates for each set of plants. Error bars indicate the standard error. **(D)** Growth phenotypes of *NtPR-Q-OE* plants and WT-Yunyan87 plants.

Differences in expression of *NtPR-Q* in response to *R. solanacearum* inoculation and the treatment of hormones suggested a role for this gene in plant immunity. To test this possibility, we established transgenic tobacco *NtPR-Q-OE* under the control of the 35S promoter in the pVCT2024 vector (**Figure 2A**). PCR analysis was used to verify the T-DNA insertion in transgenic tobacco lines (**Figure 2B**). The *NtPR-Q* transcript levels in *NtPR-Q-OE* and WT-Yunyan87 plants were determined in 10 independent transgenic T1 tobacco lines via RT-qPCR. The transgenic lines 8 and 9 exhibited higher levels of *NtPR-Q* mRNA than other *NtPR-Q-OE* lines and were therefore used for subsequent experiments (**Figure 2C**). We did not observe any differences in the growth phenotype between the *NtPR-Q-OE* transgenic plants and WT-Yunyan87 plants (**Figure 2D**).

**FIGURE 3:**
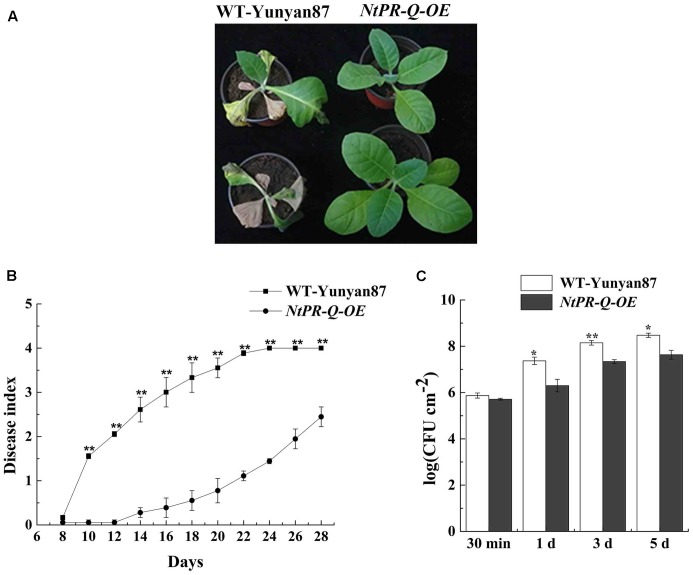
Overexpression of *NtPR-Q* enhances tobacco resistance to *R. solanacearum*. **(A)** Four-leaf-stage plants of the representative *NtPR-Q-OE* line and its parental WT-Yunyan87 15 days after irrigation with a 10 mL suspension (1 × 10^8^ CFU mL^-1^). **(B)** *R. solanacearum*-inoculated plants were scored using a disease index ranging from 0 to 4: 0 = asymptomatic plant, 1 = less than 25% leaves wilted, 2 = less than 50% wilted leaves, 3 = more than 50% wilted leaves and 4 = completely wilted leaves (dead plant). The averages presented are based on three independent biological replicates for each set of plants. **(C)** *R. solanacearum* growth assay. The amount of *R. solanacearum* in the leaves of WT-Yunyan87 and *NtPR-Q-OE* tobacco plants at 30 min, 1, 3, and 5 dpi. The leaves were inoculated with a 20 μL suspension (1 × 10^8^ CFU mL^-1^) of the highly virulent *R. solanacearum* strain CQPS-1. The averages presented are based on three independent biological replicates, each comprising three plants. Error bars indicate the standard error, Asterisks indicates a significant difference (*t*-test, ^∗^*p* < 0.05, ^∗∗^*p* < 0.01).

*NtPR-Q-OE* and WT-Yunyan87 tobacco plants were infected by highly virulent *R. solanacearum* strain CQPS-1 via root irrigation. At 15 dpi, we observed clear wilting symptoms on a majority of WT-Yunyan87 plants, whereas only a few *NtPR-Q-OE* tobacco plants exhibited slight disease symptom (**Figure 3A**). At 10, 14, 18, and 22 dpi, the disease indexes of *NtPR-Q-OE* tobacco plants were approximately 0.06, 0.28, 0.55, and 1.11, respectively, but more intense wilting symptoms were observed in the WT-Yunyan87 plants, the disease indexes were approximately 1.56, 2.61, 3.33, and 3.89, respectively (**Figure 3B**). To measure the bacterial growth, WT-Yunyan87 plants and *NtPR-Q-OE* tobacco were inoculated by 20 μL inoculum (1 × 10^8^ CFU mL^-1^) into the third leaves. The results showed that the amount of *R. solanacearum* in the leaves of WT-Yunyan87 plants was significantly higher than *NtPR-Q-OE* tobacco plants at 0, 1, 3, and 5 dpi (**Figure 3C**).

**FIGURE 4:**
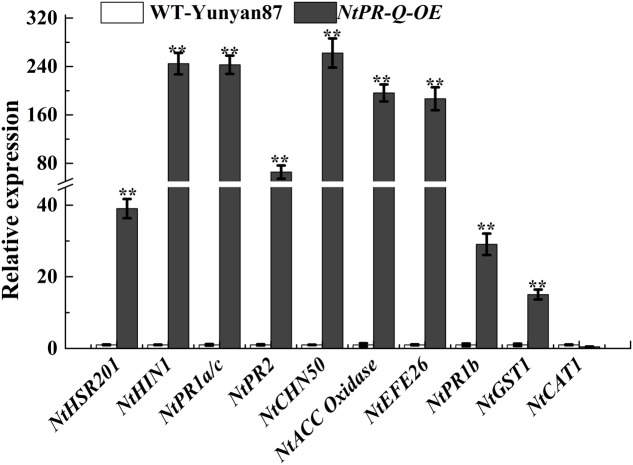
RT-qPCR analysis of relative expression of defense marker genes in *NtPR-Q-OE* plants compared with WT-Yunyan87 plants. Defense-related gene transcript levels in WT-Yunyan87 plants were used as controls, *NtUBI3* as the reference gene. Error bars indicate the standard error. The averages presented are based on three independent replicates. Asterisks indicate a significant difference (*t*-test, ^∗^*p* < 0.05, ^∗∗^*p* < 0.01).

To further affirm the role of *NtPR-Q* in disease resistance and explore the possible mechanism that how *NtPR-Q* overexpression enhanced tobacco resistance to *R. solanacearum*, the transcriptional expression of defense-related genes in *NtPR-Q-OE* plants were investigated by RT-qPCR. We examined the transcript levels of the SA-responsive genes *NtPR1a/c*, *NtPR2*, and *NtCHN50* (Brogue et al., 1991; Ward et al., 1991a), the JA-responsive gene *NtPR1b* (Sohn et al., 2007), ET production-associated genes *NtACC Oxidase* and *NtEFE26* (Chen et al., 2003), the HR-associated genes *NtHSR201* and *NtHIN1* (Sohn et al., 2007) and the reactive oxygen species (ROS) detoxification-associated genes *NtGST1* and *NtCAT1* (Takahashi and Nagata, 1992; Takahashi et al., 1997). All the genes were shown to be up-regulated in response to pathogen infection (Rizhsky et al., 2002; Sohn et al., 2007; Chen et al., 2012). The results of RT-qPCR showed that the transcript levels of these genes were significantly increased in *NtPR-Q-OE* plants except for *NtCAT1* (**Figure 4**).

**FIGURE 5:**
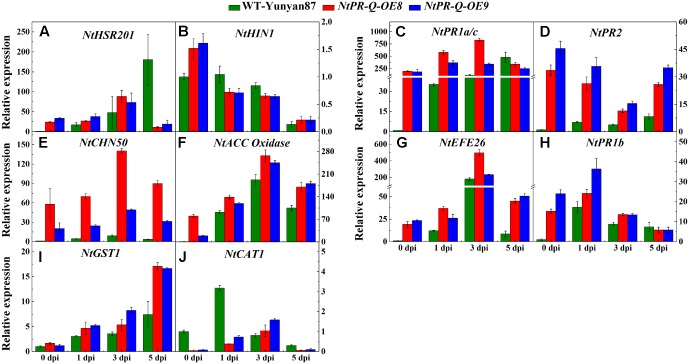
Relative expression of defense-related marker genes in *NtPR-Q-OE8, NtPR-Q-OE9* and WT-Yunyan87 tobacco plants after *R. solanacearum* inoculation. RT-qPCR analysis of relative transcript levels of defense-related genes in *NtPR-Q-OE* and WT-Yunyan87 plants at 0, 1, 3, and 5 dpi. **(A,B)** Relative transcript levels of the hypersensitive response (HR)-maker genes *NtHSR201* and *NtHIN1*. **(C–E)** Relative transcript levels of the salicylic acid (SA)-responsive genes *NtPR1a/c*, *NtPR2* and *NtCHN50*. **(F,G)** Relative transcript levels of the ethylene biosynthesis-associated genes *NtACC Oxidase* and *NtEFE26*. **(H)** Relative transcript levels of the jasmonic acid-responsive gene *NtPR1b*. **(I,J)** Relative transcript levels of the reactive oxygen species detoxification-associated genes *NtCAT1* and *NtGST1*. Plants inoculated with sterile water as control, *NtUBI3* as the reference gene. The averages presented are based on three independent biological replicates for each set of plants. Error bars indicate the standard error.

The transcriptional profiles of defense-related genes in *R. solanacearum*-infected tobacco *NtPR-Q-OE8*, *NtPR-Q-OE9*, and WT-Yunyan87 were investigated by RT-qPCR (**Figure 5**). After *R. solanacearum* infection, the transcript levels of HR- (*NtHIN1)*, SA- (*NtPR1a/c*, *NtPR2* and *NtCHN50*), ET- (*NtEFE26*, *NtACC Oxidase*), JA- (*NtPR1b*) and ROS- (*NtGST1*) associated genes in *NtPR-Q-OE* plants were higher than in WT-Yunyan87 plants at least at three of the four tested timepoints. While, expression levels of *NtHIN1* in WT-Yunyan87 and *NtPR-Q-OE* plants were similar.

### Effect of Exogenous SA, MeJA, and Ethephon Application on Disease Control

Salicylic acid, MeJA, and ethephon application led to an increase in *NtPR-Q* transcript levels (**Figure 1**). To assess the capacity of them in the resistance of tobacco to *R. solanacearum*, the disease index was surveyed after treatment with 2 mM SA, 0.1 mM MeJA, and 7 mM ethephon. The results showed that the disease index after MeJA treatment was lower than control but the difference was not significant. Tobacco plants treated with SA were significantly more resistant than the control. Ethephon application also obvious enhanced the plants resistance at 10–22 days but had no significant effect at final stage. We found that the application of these hormones enhanced the resistance of tobacco to *R. solanacearum*, with the effect of SA application being the best (**Table 2**).

**Table 2:** Control efficacy of phytohormone treatments against tobacco bacterial wilt.

Treatment	6 dpi	10 dpi	14 dpi	18 dpi	22 dpi	26 dpi
MeJA	0.21 ± 0.02^B^	0.87 ± 0.02^AB^	1.74 ± 0.03^AB^	2.45 ± 0.08^AB^	2.91 ± 0.09^AB^	3.20 ± 0.11^A^
SA	0.32 ± 0.01^B^	0.82 ± 0.19^AB^	1.23 ± 0.15^B^	1.57 ± 0.02^C^	1.77 ± 0.05^C^	2.15 ± 0.09^B^
Ethephon	0.10 ± 0.00^B^	0.57 ± 0.02^B^	1.23 ± 0.02^B^	2.12 ± 0.07^B^	2.42 ± 0.18^B^	3.17 ± 0.15^A^
CK	0.65 ± 0.14^A^	1.23 ± 0.07^A^	2.16 ± 0.19^A^	2.80 ± 0.17^A^	3.36 ± 0.12^A^	3.77 ± 0.12^A^

*Plants sprayed with sterile water served as control. Data are presented by the mean ± standard error based on three independent biological replicates. Different capital letters indicate extremely significant difference [Student–Newman–Keuls (SNK) test, *p* < 0.01]*.

## Discussion

Numerous studies have shown that the members of *PR* genes are involved in the resistance of plants to various biotic and abiotic stresses (Schultheiss et al., 2003). The expression levels of many *PR* genes are induced by many factors (Datta and Muthukrishnan, 1999), such as fungi, bacteria, and nematodes (Fujibe et al., 2000; Hamamouch et al., 2011), as well as SA, β-aminobutyric acid (Li et al., 2014), cold and drought (Gao et al., 2013; Chen et al., 2015), light (Sessa et al., 1995). Gao et al. (2013) found that the expression of *PR3* was up-regulated under salt stress. Similarly, *Xanthomonas campestris* pv. *pruni* (*Xcp*) led to the induction of peach PR transcripts, including *Pp-PR1b*, three PR5 genes *Pp-TLPA*, *Pp-TLP2*, and *Pp-TLP3*, to a significantly higher degree in the resistant cultivar (Sherif et al., 2012). In addition, overexpression of defense-related proteins, such as PR protein and WRKY transcription factor lead to the up-regulation of *PR-Q* expression level. A pepper basic PR1 gene overexpression in tobacco enhances resistance against heavy metals and pathogens *Phytophthora nicotianae*, *R. solanacearum* and *Pseudomonas syringae* pv. *tabaci* and increases the expression of *NtPR-Q* and glutathione *S*-transferase genes (Sarowar et al., 2005). The expression of *NtPR-Q* in *CaWRKY40*-overexpressing tobacco was higher than that in wild-type plants (Dang et al., 2013). We found that the expression of *NtPR-Q* was markedly induced by *R. solanacearum* attack and a variety of plant hormone treatments. Overexpression *NtPR-Q* in tobacco enhanced the resistance to *R. solanacearum* attack. PR-Q, a member of the PR-3 family, is a class II chitinase. Plant chitinase were previously shown to be involved in the resistance of plant to fungi and harmful insects (Zhu et al., 1994; Golshani et al., 2015). In this paper, *NtPR-Q* as a tobacco chitinase was found to be functional in plant resistance to *R. solanacearum* infection.

The transcript accumulation of HR-associated genes and *PR* genes is typically observed during defense responses and serves as a marker for this type of biological condition. The enhanced resistance of *NtPR-Q-OE* plants was associated with the enhanced expression of defense-associated genes, such as the HR-associated genes *NtHSR201* and *NtHIN1*, the SA-responsive genes *NtPR1a/c*, *NtPR2*, and *NtCHN50*, ET production-associated genes such as *NtACC Oxidase* and *NtEFE26*, the JA-responsive gene *NtPR1b* and the reactive oxygen species (ROS) detoxification-associated gene *NtGST1*. Thus, *NtPR-Q*, a PR protein, promotes the transcription levels of a range of HR- and defense-associated genes, resulting in enhancing tobacco resistance against *R. solanacearum.*

Salicylic acid, JA, and ET have been shown to activate the *PR* genes expression of plants in varying degrees and act either synergistically (Devadas et al., 2002; Mur et al., 2006) or antagonistically (Kunkel and Brooks, 2002) during defense signaling, dependent on their concentrations. In peach, *Pp-PR1a* was induced by SA, whereas the *Pp-PR1b*, *Pp-TLPA*, *Pp-TLP2*, and *Pp-TLP3* genes were induced mainly by ET or MeJA treatments. The induction of the same set of PR genes in response to pathogen infection, MeJA or ethephon treatment suggests the involvement of JA/ethylene-signaling pathways in mediating resistance against *Xcp* (Sherif et al., 2012). In this paper, *NtPR-Q* was induced by SA, MeJA and ethephon application, and the overexpression of *NtPR-Q* enhanced the expression of SA-dependent *NtPR1a/c*, *NtPR2*, and *NtCHN50*, JA-responsive *NtPR1b* and ET associated genes *NtACC Oxidase* and *NtEFE26* by *R. solanacearum* infection. These results suggest that *NtPR-Q* might influence SA, JA and ET-mediated defense signaling during *R. solanacearum* infection. Although there were a lot of studies to explore the role of chitinase in plant disease resistance, it only tested the plant resistance to fungi pathogens and the expression of other PR genes was not analyzed (Zhu et al., 1994; Jongedijk, 1995). Our RT-qPCR analysis showed that the *NtPR-Q* overexpression enhanced the expression of a whole range of defense-related genes, which seems to support the conclusion that NtPR-Q induces plant SAR. We speculate that the constitutive expression of *PR-Q* results in the accumulation of unfolded proteins in the endoplasmic reticulum (ER), which leads to ER stress and activates defense signal pathways, thereby enhances the resistance of plants to *R. solanacearum*. This phenomenon is referred as unfolded protein response (UPR) (Chakraborty et al., 2016; Wan and Jiang, 2016). However, to verify this hypothesis, we still need further evidence.

## Conclusion

It was concluded that tobacco plants exhibited resistance to bacterial wilt induced by *R. solanacearum* when *NtPR-Q* was constitutively expressed, which indicates potency of *NtPR-Q* in plant resistance breeding. However, it is still needs further study on the specific biological functions of *NtPR-Q* and its involvement in the resistance to other environmental stresses.

## Author Contributions

The experiment was designed by WD and QL. YT, QL, and YL accomplished the experiments. YT analyzed the data. YT and WD wrote the paper, and the paper was revised by YT, QL, and LZ.

## Conflict of Interest Statement

The authors declare that the research was conducted in the absence of any commercial or financial relationships that could be construed as a potential conflict of interest.

## References

[B1] Agrios G. N. (1988). “How pathogens attack plants,” in *Plant Pathology* (Elsevier: Academic Press), 63–86.

[B2] Brogue K. Chet I. Holliday M. Cressman R. Biddle P. Knowlton S. (1991). Transgenic plants with enhanced resistance to the fungal pathogen *Rhizoctonia solani*. *Science* 254 1194–1197. 10.1126/science.254.5035.1194 17776411

[B3] Chakraborty R. Ji H. B. Bae E. Y. Kim W.-Y. Lee S. Y. Kim M. G. (2016). Comparison and contrast of plant, yeast, and mammalian ER stress and UPR. *Appl. Biol. Chem.* 59 337–347. 10.1007/s13765-016-0167-6

[B4] Chen L. Yu S. Li S. Zhang L. Zou C. Yu D. (2012). The role of WRKY transcription factors in plant abiotic stresses. *Biochim. Biophys. Acta* 1819 120–128. 10.1016/j.bbagrm.2011.09.002 21964328

[B5] Chen L. J. Ren H. Deng X. G. Li Y. N. Cha W. Q. Lin H. H. (2015). Effects of light intensity on the susceptibility of *Nicotiana tabacum* to cucumber mosaic virus. *J. Gen. Plant Pathol.* 81 399–408. 10.1007/s10327-015-0602-2

[B6] Chen N. Goodwin P. H. Hsiang T. (2003). The role of ethylene during the infection of *Nicotiana tabacum* by *Colletotrichum destructivum*. *J. Exp. Bot.* 54 2449–2456. 10.1093/jxb/erg289 14565949

[B7] Dang F. F. Wang Y. N. Lu Y. U. Eulgem T. Lai Y. Liu Z. Q. (2013). CaWRKY40, a WRKY protein of pepper, plays an important role in the regulation of tolerance to heat stress and resistance to *Ralstonia solanacearum* infection. *Plant Cell Environ.* 36 757–774. 10.1111/pce.12011 22994555

[B8] Datta S. K. Muthukrishnan S. (1999). *Pathogenesis-Related Proteins of Plants.* Boca Raton, FL: CRC Press, 21–47.

[B9] Devadas S. K. Enyedi A. Raina R. (2002). The *Arabidopsis hrl1*, mutation reveals novel overlapping roles for salicylic acid, jasmonic acid and ethylene signalling in cell death and defence against pathogens. *Plant J.* 30 467–480. 10.1046/j.1365-313X.2002.01300.x 12028576

[B10] Fujibe T. Watanabe K. Nakajima N. Ohashi Y. Mitsuhara I. Yamamoto K. T. (2000). Accumulation of pathogenesis-related proteins in tobacco leaves irradiated with UV-B. *J. Plant Res.* 113 387–394. 10.1007/PL00013946

[B11] Fujita M. Fujita Y. Noutoshi Y. Takahashi F. Narusaka Y. Yamaguchi-Shinozaki K. (2006). Crosstalk between abiotic and biotic stress responses: a current view from the points of convergence in the stress signaling networks. *Curr. Opin. Plant Biol.* 9 436–442. 10.1016/j.pbi.2006.05.014 16759898

[B12] Gao Q. Zeng X. Jia L. Niu D. Li X. Guan M. (2013). The expression profiling of rice pathogenesis-related proteins in seedling stage under environmental stresses. *Prog. Biochem. Biophys.* 40 1140 10.3724/SP.J.1206.2013.00014

[B13] Glazebrook J. (2005). Contrasting mechanisms of defense against biotrophic and necrotrophic pathogens. *Annu. Rev. Phytopathol.* 43 205–207. 10.1146/annurev.phyto.43.040204.135923 16078883

[B14] Golshani F. Fakheri B. A. Behshad E. Vashvaei R. M. (2015). PRs proteins and their mechanism in plants. *Biol. Forum Int. J.* 7 477–495.

[B15] Hamamouch N. Li C. Seo P. J. Park C. M. Davis E. L. (2011). Expression of *Arabidopsis* pathogenesis-related genes during nematode infection. *Mol. Plant Pathol.* 12 355–364. 10.1111/j.1364-3703.2010.00675.x 21453430 PMC6640486

[B16] Horita M. Suga Y. Ooshiro A. Tsuchiya K. (2010). Analysis of genetic and biological characters of Japanese potato strains of *Ralstonia solanacearum*. *J. Gen. Plant Pathol.* 76 196–207. 10.1007/s10327-010-0229-2

[B17] Ji P. Momol M. T. Olson S. M. Pradhanang P. M. Jones J. B. (2005). Evaluation of thymol as biofumigant for control of bacterial wilt of tomato under field conditions. *Plant Dis.* 89 497–500. 10.1094/PD-89-0497 30795428

[B18] Jia H. Wang C. Wang F. Liu S. Li G. Guo X. (2015). *GhWRKY68* reduces resistance to salt and drought in transgenic *Nicotiana benthamiana*. *PLOS ONE* 10:e0120646. 10.1371/journal.pone.0120646 25793865 PMC4368093

[B19] Jones J. D. G. Dangl J. L. (2006). The plant immune system. *Nature* 444 323–329. 10.1038/nature05286 17108957

[B20] Jongedijk E. (1995). Synergistic activity of chitinases and β-1,3-glucanases enhances fungal resistance in transgenic tomato plants. *Euphytica* 85 173–180. 10.1007/BF00023946

[B21] Kessmann H. Staub T. Ligon J. Oostendoro M. Ryals J. (1994). Activation of systemic acquired disease resistance in plants. *Eur. J. Plant Pathol.* 100 359–369. 10.1007/BF01874804

[B22] Kunkel B. N. Brooks D. M. (2002). Cross talk between signaling pathways in pathogen defense. *Curr. Opin. Plant Biol.* 5 325–331. 10.1016/S1369-5266(02)00275-3 12179966

[B23] Kushalappa A. C. Yogendra K. N. Karre S. (2016). Plant innate immune response: qualitative and quantitative resistance. *Crit. Rev. Plant Sci.* 35 38–55. 10.1080/07352689.2016.1148980

[B24] Li S. Xu C. Wang J. Guo B. Yang L. Chen J. (2017). Cinnamic, myristic and fumaric acids in tobacco root exudates induce the infection of plants by *Ralstonia solanacearum*. *Plant Soil* 412 381–395. 10.1007/s11104-016-3060-5

[B25] Li X. Wei F. Niu D. Guan M. Miao L. Shi J. (2014). Expression analysis of pathogenesis-related 1 proteins in normal growth of rice leaves and interactions between rice and *Xanthomonas oryzae pv.oryzae*. *Chin. Bull. Bot.* 49 127–138. 10.3724/SP.J.1259.2014.00127 23234404

[B26] Liu Y. Tang Y. Qin X. Yang L. Jiang G. Li S. (2017a). Genome sequencing of *Ralstonia solanacearum* CQPS-1, a phylotype I strain collected from a highland area with continuous cropping of tobacco. *Front. Microbiol.* 8:974. 10.3389/fmicb.2017.00974 28620361 PMC5449461

[B27] Liu Y. Wu D. Liu Q. Zhang S. Tang Y. Jiang G. (2017b). The sequevar distribution of *Ralstonia solanacearum*, in tobacco-growing zones of China is structured by elevation. *Eur. J. Plant Pathol.* 147 541–551. 10.1007/s10658-016-1023-6

[B28] Mur L. A. Kenton P. Atzorn R. Miersch O. Wasternack C. (2006). The outcomes of concentration-specific interactions between salicylate and jasmonate signaling include synergy, antagonism, and oxidative stress leading to cell death. *Plant Physiol.* 140 249–262. 10.1104/pp.105.072348 16377744 PMC1326048

[B29] Murashige T. Skoog F. (1962). A revised medium for rapid growth and bio assays with tobacco tissue cultures. *Physiol. Plant.* 15 473–497. 10.1111/j.1399-3054.1962.tb08052.x

[B30] Oh S. K. Park J. M. Joung Y. H. Lee S. Chung E. Kim S. Y. (2005). A plant EPF-type zinc-finger protein, CaPIF1, involved in defence against pathogens. *Mol. Plant Pathol.* 6 269–285. 10.1111/j.1364-3703.2005.00284.x 20565656

[B31] Ohme-Takagi M. Jr. Meins F. Shinshi H. (1998). A tobacco gene encoding a novel basic class II chitinase: a putative ancestor of basic class I and acidic class II chitinase genes. *Mol. Genet. Genomics* 259 511–515. 10.1007/s004380050842 9790582

[B32] Payne G. Ahl P. Moyer M. Harper A. Beck J. Meins F. (1990). Isolation of complementary DNA clones encoding pathogenesis-related proteins P and Q, two acidic chitinases from tobacco. *Proc. Natl. Acad. Sci. U.S.A.* 87 98–102. 10.1073/pnas.87.1.98 2296608 PMC53207

[B33] Prior P. Fegan M. (2005). Recent development in the phylogeny and classification of *Ralstonia solanacearum*. *Acta Hortic.* 695 127–136. 10.17660/ActaHortic.2005.695.14

[B34] Rizhsky L. Liang H. Mittler R. (2002). The combined effect of drought stress and heat shock on gene expression in tobacco. *Plant Physiol.* 130 1143–1151. 10.1104/pp.006858 12427981 PMC166635

[B35] Safavi K. Zareie R. (2012). Constitutive expression of thaumatin-like protein (TLP-3) in transgenic tobacco plants leads to enhance resistance to *Alternaria alternata*. *Arch. Phytopathol. Plant Prot.* 45 161–169. 10.1080/03235408.2010.507947

[B36] Sarowar S. Kim Y. J. Kim E. N. Kim K. D. Hwang B. K. Islam R. (2005). Overexpression of a pepper basic pathogenesis-related protein 1 gene in tobacco plants enhances resistance to heavy metal and pathogen stresses. *Plant Cell Rep.* 24 216–224. 10.1007/s00299-005-0928-x 15719238

[B37] Schultheiss H. Dechert C. Király L. Fodor J. Michel K. Kogel K. H. (2003). Functional assessment of the pathogenesis-related protein PR-1b in barley. *Plant Sci.* 165 1275–1280. 10.1016/S0168-9452(03)00336-4

[B38] Sels J. Mathys J. De Coninck B. M. Cammue B. P. De Bolle MF (2008). Plant pathogenesis-related (PR) proteins: a focus on PR peptides. *Plant Physiol. Biochem.* 46 941–950. 10.1016/j.plaphy.2008.06.011 18674922

[B39] Sessa G. Yang X. Q. Raz V. Eyal Y. Fluhr R. (1995). Dark induction and subcellular localization of the pathogenesis-related PRB-1b protein. *Plant Mol. Biol.* 28 537–547. 10.1007/BF00020400 7632922

[B40] Sherif S. Paliyath G. Jayasankar S. (2012). Molecular characterization of peach PR genes and their induction kinetics in response to bacterial infection and signaling molecules. *Plant Cell Rep.* 31 697–711. 10.1007/s00299-011-1188-6 22101723

[B41] Sohn S. I. Kim Y. H. Kim B. R. Lee S. Y. Lim C. K. Hur J. H. (2007). Transgenic tobacco expressing the *hrpN(EP)* gene from *Erwinia pyrifoliae* triggers defense responses against *Botrytis cinerea*. *Mol. Cells* 24 232–239. 17978576

[B42] Takahashi H. Chen Z. Du H. Liu Y. Klessig D. F. (1997). Development of necrosis and activation of disease resistance in transgenic tobacco plants with severely reduced catalase levels. *Plant J.* 11 993–1005. 10.1046/j.1365-313X.1997.11050993.x 9193071

[B43] Takahashi Y. Nagata T. (1992). *parB*: an auxin-regulated gene encoding glutathione S-transferase. *Proc. Natl. Acad. Sci. U.S.A.* 89 56–59. 10.1073/pnas.89.1.56 PMC48174 1729717

[B44] Uknes S. Mauch-Mani B. Moyer M. Potter S. Williams S. Dincher S. (1992). Acquired resistance in arabidopsis. *Plant Cell* 4 645–656. 10.2307/3869523 1392589 PMC160161

[B45] Vlot A. C. Dempsey D. A. Klessig D. F. (2009). Salicylic acid, a multifaceted hormone to combat disease. *Annu. Rev. Phytopathol.* 47 177–206. 10.1146/annurev.phyto.050908.135202 19400653

[B46] Wan S. Jiang L. (2016). Endoplasmic reticulum (ER) stress and the unfolded protein response (UPR) in plants. *Protoplasma* 253 753–764. 10.1007/s00709-015-0852-z 26060134

[B47] Wang Y. Dang F. Liu Z. Wang X. Eulgem T. Lai Y. (2013). *CaWRKY58*, encoding a group I WRKY transcription factor of *Capsicum annuum*, negatively regulates resistance to *Ralstonia solanacearum* infection. *Mol. Plant Pathol.* 14 131–144. 10.1111/j.1364-3703.2012.00836.x 23057972 PMC6638745

[B48] Ward E. R. Payne G. B. Moyer M. B. Williams S. C. Dincher S. S. Sharkey K. C. (1991a). Differential regulation of beta-1, 3-glucanase messenger RNAs in response to pathogen infection. *Plant Physiol.* 96 390–397. 10.1104/pp.96.2.390 16668198 PMC1080782

[B49] Ward E. R. Uknes S. J. Williams S. C. Dincher S. S. Wiederhold D. L. Alexander D. C. (1991b). Coordinate gene activity in response to agents that induce systemic acquired resistance. *Plant Cell* 3 1085–1094. 10.2307/3869297 12324583 PMC160074

[B50] Yang L. Ding W. Xu Y. Wu D. Li S. Chen J. (2016). New insights into the antibacterial activity of hydroxycoumarins against *Ralstonia solanacearum*. *Molecules* 21:468. 10.3390/molecules21040468 27070570 PMC6273506

[B51] Zhao D. Y. Shen L. Fan B. Yu M. Zheng Y. Lv S. (2009). Ethylene and cold participate in the regulation of *LeCBF1* gene expression in postharvest tomato fruits. *FEBS Lett.* 583 3329–3334. 10.1016/j.febslet.2009.09.029 19766636

[B52] Zhu Q. Maher E. A. Masoud S. Dixon R. A. Lamb C. J. (1994). Enhanced protection against fungal attack by constitutive co-expression of chitinase and glucanase genes in transgenic tobacco. *Nat. Biotechnol.* 12 807–812. 10.1038/nbt0894-807

